# Future climate change will drive expansion of suitable planting areas for Fructus Aurantii in Jiangxi Province, China

**DOI:** 10.3389/fpls.2025.1579546

**Published:** 2025-06-18

**Authors:** Lin Chen, Xi Guo, Hengyu Zou, Anfan Zhu, Xingyu Huang

**Affiliations:** ^1^ Key Laboratory of Farmland Improvement and Quality Enhancement, Jiangxi Provincial Department of Science and Technology, Nanchang, China; ^2^ Faculty of Land Resources and Environment, Jiangxi Agricultural University, Nanchang, China; ^3^ Jiangxi Agricultural Technology Extension Center, Provincial Department of Agriculture and Rural Affairs, Nanchang, China

**Keywords:** Fructus aurantii, future global warming, Southeastern China, MAXENT model, planting suitability

## Abstract

Fructus Aurantii (FA) is a valuable medicinal material used in traditional China medicine. Predicting the suitable distribution areas of FA and identifying its potential distribution patterns driven by various environmental factors are crucial for the selection of planting sites and maintenance of medicinal quality. Here, the maximum entropy model was used to predict the potential distribution of FA in Jiangxi Province, China under current and future climate conditions. A total of 105 geographical distribution data of FA were collected through field investigation and 32 environmental variables were obtained from public databases. The maximum entropy model showed high prediction accuracy when 16 environmental variables were selected (AUC = 0.932). The habitat suitability of FA was prominently affected by climate, which surpassed topography and soil factors. Maximum temperature of the warmest month, annual temperature range, precipitation of the wettest month, precipitation coefficient of variation, elevation, aspect, and soil organic carbon were the key factors shaping the geographic distribution of FA. Among them, maximum temperature of the warmest month (16.9%), followed by annual temperature range (16.1%), made the greatest contribution to model predictions. In the current climate background, the total potential suitable area for FA covered 6.30 × 10^4^ km^2^ of garden land. Under future climate warming scenarios (shared socioeconomic pathways 245, 585), the potential suitable area was predicted to move southward and expand twice in 2040–2080, with notable increase in moderately and poorly suitable areas. Low hilly areas at higher elevations with moist cool conditions and gentle undulations would become more suitable for future introduction and planting of FA. Regionalized strategies for different suitable planting areas were proposed taking into account future climate change. All data are available in Mendeley Data (DOI: 10.17632/s9wsnn2xcn.1). Code is available at https://github.com/mrmaxent/Maxent.

## 1 Introduction

Fructus Aurantii (FA) is the dried unripe fruit of *Citrus aurantimu* L. and its cultivars. The use of FA in traditional Chinese medicine can be dated back to the Tang Dynasty in China. As a precious Chinese medicinal material, FA plays a role in regulating qi flowing to smooth the middle burner and resolving food stagnation to relieve abdominal distension (Pharmacopoeia Commission of the People's Republic of China, 2010). Based on the place of origin, there are six major categories of FA: Jiang FA from Jiangxi, Xiang FA from Hunan, Chuan FA from Sichuan, Su FA from Jiangsu, Wen FA from Wenzhou, and Qu FA from Quzhou. Among these, Jiang FA, Xiang FA, and Chuan FA are most popular, accounting for >70% of the total FA production in China. Jiang FA (hereinafter referred to as FA) has been cultivated for over 1700 years, with Zhangshu City and Xingan County as the primary genuine production areas. The FA derived from *C. aurantium* ‘Xiucheng’ and *C. Junos* is considered to have high quality nationwide (Xie B. et al., 2024). However, due to the multi-source and multi-origin of Chinese medicinal materials, their product quality (particularly the content of effective ingredients) varies across regions. The quality stability and uniformity of Chinese medicinal materials are easily compromised by the mixing of plant sources. Therefore, identifying the suitable distribution range of FA is crucial for its introduction, domestication, and conservation on a regional scale.

As a key external factor affecting the quality of genuine medicinal materials, the geographical environment directly controls plant growth and development, as well as the formation and accumulation of effective ingredients (Zhong et al., 2013). To date, climate warming has become a global trend. In the Sixth Assessment Report, the Intergovernmental Panel on Climate Change (IPCC) estimated that the global average surface temperature during 2000–2020 increased by 1.1°C compared to the preindustrial levels during 1850–1900. Models projections indicated that global warming would reach 1.5°C above the preindustrial levels in 2040, and even reach 2.7–4.8°C by 2081–2100 under moderate and high CO_2_ emission scenarios (IPCC, 2021). In the face of future climate warming, the production areas of FA are likely to undergo dramatical environmental changes. Heat stress can shorten plant growth cycle, resulting in poor fruit development or premature ripening (Oyedoh et al., 2024). Additionally, global warming has increased the frequency and intensity of climate extremes (e.g., typhoons, droughts, floods), interfering with plant normal growth (Fan et al., 2024). The environmental changes potentially alter the structure and composition of biological communities and destroy the balance and stability of the original ecosystem, affecting the quality of FA (Wang et al., 2024). In this case, the conventional planting areas for FA are expected to lose their advantages, with other places emerging as the new suitable planting areas. Therefore, re-evaluating the distribution of suitable planting areas for FA under future climate change can provide guidance on its scientific introduction and expansion, ensuring high product quality.

Accurate prediction of species distribution and habitat suitability is a major task of ecological research, which paves the way for biodiversity conservation, resource management, and sustainable ecosystem development. The advent of geographic information systems, together with digital-elevation-model-based terrain analysis and non-parametric statistical analysis, allows species distribution models to be extensively used in spatial ecology (Gelfand, 2020). Such models have been applied in predicting the spatiotemporal distribution patterns of aquatic species (Chen et al., 2023) and the potential distribution areas of invasive species (Mushtaq et al., 2021), rare and endangered species (Majeed et al., 2023), and plant diseases and pests (Ikegami and Jenkins, 2018; Xian et al., 2023). To illustrate, Safei et al. (2018) used logistic regression (LR), non-parametric multiplicative regression (NPMR), and ecological-niche factor analysis (ENFA) methods to generate the potential distribution maps of Astragalus verus Olivier in the semi-arid region of central Iran. Rawat et al. (2022) modeled the current and future potential habitat distribution of the endangered medicinal plant Picrorhiza kurroa (Royle ex Benth) in the Uttarakhand Himalaya region using the maximum entropy (MaxEnt) model. Based on comprehensive analysis and utilization, the maximum entropy model (MaxEnt) has proven superior to ecological niche models represented by the bioclimate analysis and prediction system (BIOCLIM) (Serrano-Notivoli et al., 2022), the ecological niche factor analysis model (ENFA) (Farashi et al., 2013), and the genetic algorithm for rule-set prediction (GARP) (Hu et al., 2020). MaxEnt is advantageous in terms of fewer species distribution points required, simpler modeling procedures, higher prediction accuracy, and easier data interpretation, as well as relatively objective and reasonable evaluation results. This model has been applied increasingly in many research fields, including biogeography and conservation biology (He et al., 2021; Rong et al., 2023; Wu et al., 2024; Xie M. et al., 2024; Zou et al., 2024).

The MaxEnt model is based on the principle of MaxEnt in information theory proposed by Jaynes in 1957 (Jaynes, 1957). The core idea of the MaxEnt principle is that the probability distribution that maximizes the entropy is closest to the true state when partial information is known. The ecological regionalization of Chinese medicinal materials emphasizes the concept of “place of origin”; that is, high-quality Chinese medicinal materials can be produced only when source plants are grown in areas with a highly similar ecological environment to the place of origin (Xie et al., 2016). If the species distribution and relevant environmental variables are known, the MaxEnt model can use this limited information to predict the potential distribution areas of species under different environmental conditions by constructing a reasonable probability distribution. This model has outstanding robustness under future changing scenarios (Shi et al., 2023; Zhao et al., 2021, 2024). While FA production is plagued by problems such as the confusion of plant sources and nonuniform quality, the MaxEnt Model provides crucial technical support for planting expansion, stable production, artificial cultivation, and resource conservation of FA.

In this study, the MaxEnt model was used to predict the potential distribution of suitable planting areas for FA in Jiangxi Province under future climate change. We aimed to clarify the future suitable distribution range of FA, allowing for rational planning of planting areas and scientific formulation of resource management strategies. The results could have important implications for the sustainable development of regional FA industry, ensuring ecosystem balance and stability.

## 2 Materials and methods

### 2.1 Study area

**Figure 1:**
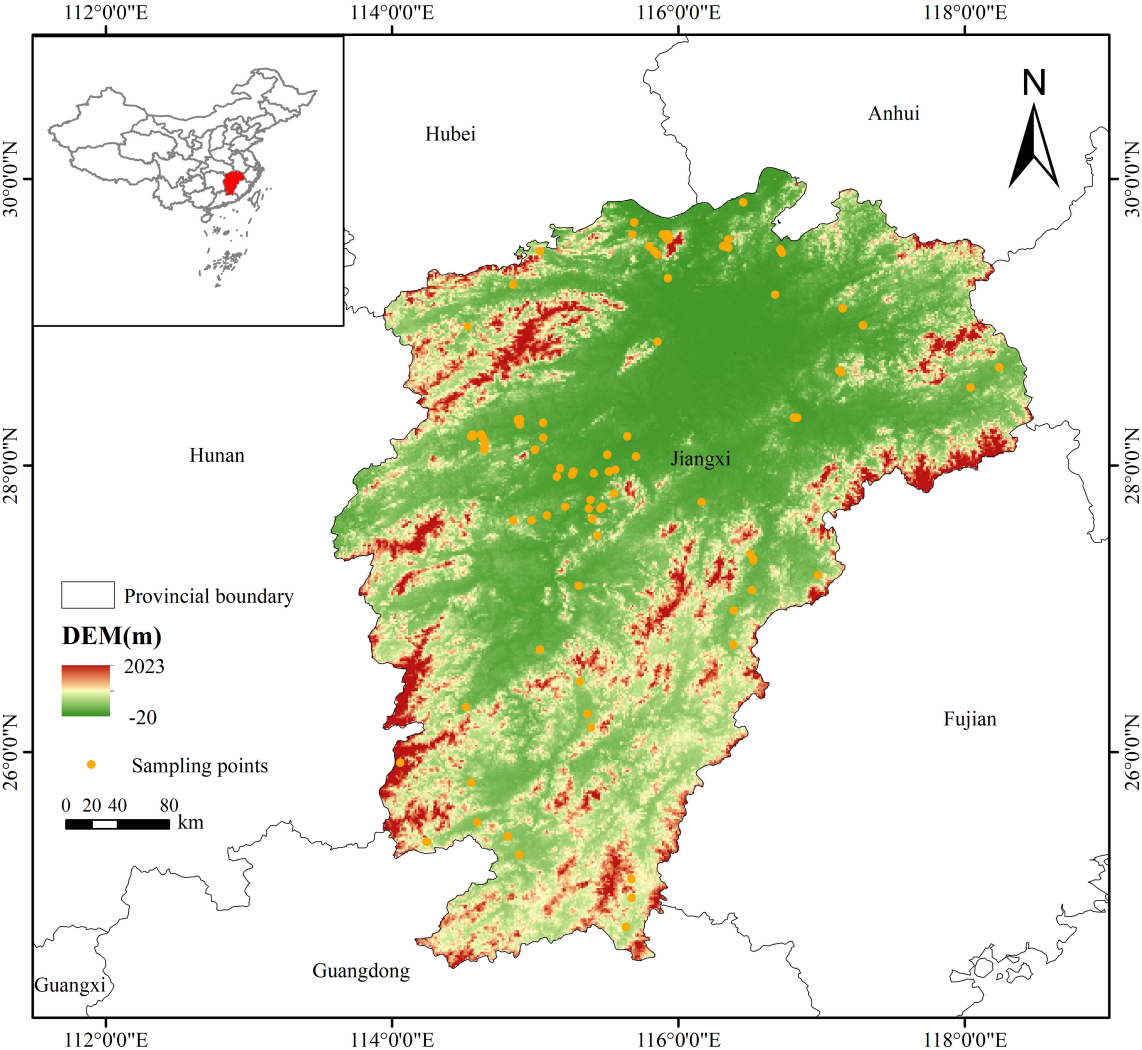
Location of Jiangxi Province in southern China and distribution of Fructus Aurantii sampling points in the study area.

Jiangxi Province is located in the southeastern region of China, between the geographical coordinates of 24°29’–30°04’ N and 113°34’–118°28’ E (**Figure 1**). This region mainly encompasses hilly and mountainous areas, including the Poyang Lake Plain and other landforms. The terrain is high in the south and low in the north. Based on its macro-geographical pattern, Jiangxi can be roughly divided into five parts, covering a total area of 16.69×10^4^ km^2^ (Jiang et al., 2020). The Poyang Lake Plain area mainly includes Nanchang, Jiujiang, and Jingdezhen, with elevations mostly below 50 m above sea level (asl). The northwestern mountainous area is primarily composed of Yichun and Pingxiang, and the northeastern mountainous area mainly includes Shangrao and Yingtan, both of which have an average elevation of ~300 m asl. The central hilly area primarily covers Ji’an, Fuzhou, and Xinyu, with an average elevation of 200 m asl. The southern mountainous area is mainly located in Ganzhou, with an average elevation of 400 m asl.

Jiangxi experiences various climate types, and the subtropical monsoon climate prevailing in this region is characterized by four distinct seasons with abundant rainfall. The average annual temperature is between 16.3–25°C. The winter is warm and the summer is hot, with a frost-free period of 240–307 days. The duration of average daily temperature > 10°C is 240–270 days and the active accumulated temperature is 5000–6000°C, favorable for the development of subtropical economic trees. As one of the rainiest provinces in China, Jiangxi receives an average annual precipitation of 1341–1943 mm. The regional distribution of precipitation trends upward toward the south from the north, and more precipitation occurs in the east than in the west. In 2021, FA was certified for implementing the protection of geographical indication products (Jiangxi Provincial Market Supervision Administration, 2021), and there was a steady increase in the planting area for FA. In 2024, the government of Zhangshu proposed to build a high-quality science and technology complex for Chinese medicinal materials. An excellent germplasm resource preservation nursery and breeding base for high-quality FA seedlings was constructed, promoting the establishment of a planting area of >400,002 m^2^ (Zhangshu Bureau of Agricultural and Rural Affairs, 2024). Thus, strong policy support is provided for the planting area expansion of FA.

### 2.2 Research techniques and ideas

This study was aimed to address the following three objectives: i) evaluating the suitable ranges and relative importance of different environmental variables for FA distribution using the MaxEnt model; ii) identifying the potential distribution patterns of FA under two future shared socioeconomic pathway scenarios (SSP245, SSP585) for 2040–2080; and iii) guiding the layout of planting areas for FA based on its future potential distribution.

The research methodology consisted of the following steps: (i) collect the geographical distribution data of FA and associated environmental data in Jiangxi; (ii) preprocess and select the FA distribution data and associated environmental variables; (iii) construct the training and testing datasets; (iv) establish a potential distribution model; (v) determine suitable environmental ranges for FA; (vi) draw and compare the potential distribution maps of FA under different climate scenarios; and (vii) conduct spatial distribution analysis and planting regionalization of FA on garden land across Jiangxi.

In the step vii, the cultivation zones are classified into three distinct categories based on FA suitability assessments: core demonstration area, stable production and promotion area, potential improvement area. The core demonstration area represents areas where the ecological environment exhibits optimal alignment with FA’s physiological requirements, consistently sustaining premium quality and high-yield cultivation. The stable production and promotion area comprises regions with baseline environmental compatibility for FA growth, where yield optimization can be achieved through targeted agricultural interventions. The potential improvement area identifies localities demonstrating significant disparity between natural conditions and FA cultivation prerequisites, characterized by suboptimal productivity levels necessitating either enhanced infrastructural investments or implementation of adaptive cultivar variants to improve agricultural outcomes.

### 2.3 Collection and preprocessing of Fructus Aurantii distribution data

**Figure 2:**
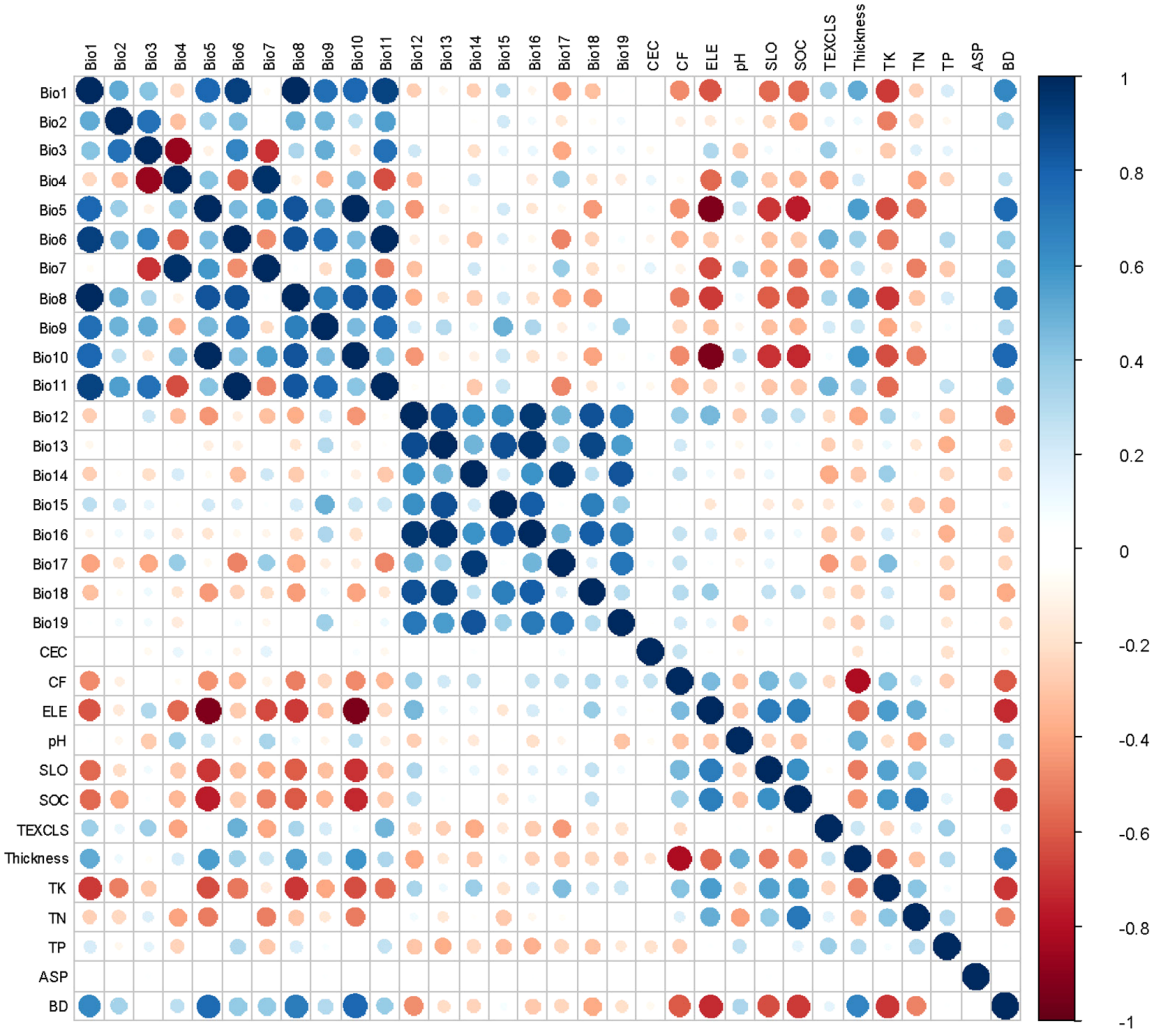
Correlation heatmap of 32 environmental variables affecting the distribution of Fructus Aurantii. The definition of all variables is provided in **Table 2**.

The geographical distribution data of FA in Jiangxi were collected from three sources, i.e., field investigation and sampling (**Figure 2**), the provincial monitoring data of Chinese medicinal materials, and China Virtual Herbarium (https://www.cvh.ac.cn/). A total of 105 FA samples were obtained in 2000-2024. Considering the spatial autocorrelation of the sampling points (Halvorsen et al., 2016), we used ENMtools (Warren et al., 2010) to eliminate autocorrelation. Only one distribution point was retained in each grid (1 km × 1 km), and 93 effective distribution points were obtained (DOI: 10.17632/s9wsnn2xcn.1).

### 2.4 Acquisition and preprocessing of environmental data

The suitability evaluation of FA requires comprehensive consideration of three key environmental factors: climatic conditions that regulate hydrothermal availability and phenological development, topographic features in Jiangxi’s mountainous terrain that create microhabitat variations through differential light and water distribution, and soil properties that determine nutrient supply and rooting conditions, collectively influencing its spatial distribution and physiological performance. Nineteen bioclimatic variables (Bio1–Bio19) and three topographic variables [elevation (ELE), slope (SLO), aspect (ASP)] were obtained from the WorldClim database (https://www.worldclim.org/). Ten soil variables [pH, soil organic carbon (SOC), bulk density (BD), cation exchange capacity (CEC), total nitrogen (TN), total phosphorus (TP), total potassium (TK), coarse fragments (CF), texture class (TEXCLS), thickness] were derived from the Soil Sub-Center, National Earth System Science Data Center, National Science & Technology Infrastructure of China (https://soil.geodata.cn/). We prepared all layers of the environment variables in TIF format (raster data) with a spatial resolution of 30” (~1 km). The geographical coordinate system was WGS84 (Gong et al., 2022).

The future climate data (2040–2060, 2060–2080) were acquired from the WorldClim database (https://www.worldclim.org/). The future climate scenario data were selected from the BCC-CSM2-MR model, which is the latest medium-resolution climate system model developed by the National Climate Center of the China Meteorological Administration. This model shows significant improvements over previous models in simulating climate variability at different time scales, long-term trends in surface air temperature, and average annual precipitation distribution in China. Notably, it exhibits particularly high accuracy in simulating extreme precipitation events in eastern China, making it well-suited for projecting future climate change scenarios across the country (Gong et al., 2017; Li S. et al., 2023; Xin et al., 2019). According to the Shared Socioeconomic Pathways (SSP) framework:SSP1-2.6 represents a sustainable development pathway with low carbon emissions; SSP2-4.5 reflects moderate emissions under balanced economic growth; SSP3-7.0 signifies a fossil fuel-dependent development pattern amid intensified regional competition; while SSP5-8.5 constitutes an extreme high-emission scenario with maximum radiative forcing (Li et al., 2020; Liu et al., 2024). This study strategically focuses on comparing the cultivation suitability of FA between representative baseline (SSP2-4.5) and extreme scenarios (SSP5-8.5). By excluding the overly optimistic SSP1-2.6 and the socioeconomic volatility-associated SSP3-7.0, our dual-scenario approach effectively captures both evolutionary patterns under conventional development trajectories and early warning signals of potential climate extremes. Many environmental factors are correlated to a certain degree. To avoid overfitting of the MaxEnt model (Han et al., 2025), the 32 raster environment variables were converted into ASCII format using ArcGIS 10.7 (ESRI, Redlands, CA, USA). Maxent 3.4.4 (Phillips et al., 2006) was used to preprocess the data of 93 FA distribution points and 32 environmental variables, and the percentage contribution of each environmental variable was calculated (**Table 1**). A total of 12 environmental variables with a cumulative contribution ≥ 80% to FA distribution were selected. These variables were maximum temperature of the warmest month (Bio5), annual temperature range (Bio7), elevation, variance of temperature change (Bio4), precipitation of the wettest month (Bio13), ASP, precipitation coefficient of variation (Bio15), CF, SOC, mean temperature of the warmest quarter (Bio10), TN, and isothermality (Bio3).

**Table 1:** Percentage contribution and permutation importance of all 32 environmental variables to the distribution of Fructus Aurantii in Jiangxi Province.

Variable		Percentage contribution (%)	Permutation importance (%)
Bio5	Maximum temperature of the warmest month	19.4	23.3
Bio7	Annual temperature range	11.2	4
ELE	Elevation	10.2	7.6
Bio4	Variance of temperature change	8.2	0
Bio13	Precipitation of the wettest month	6.9	8.1
ASP	Aspect	6.7	2.8
Bio15	Precipitation coefficient of variation	5	10.4
CF	Coarse fragments	3.3	3.3
SOC	Soil organic carbon	3	4.8
Bio10	Mean temperature of the warmest quarter	2.7	0.7
TN	Total nitrogen	2.7	0.4
Bio3	Isothermality	2.3	3
Bio19	Precipitation of the coldest quarter	2.2	6.1
SLO	Slope	2.1	1.4
pH	pH	1.6	2.2
Bio18	Precipitation of the warmest quarter	1.5	1.8
Bio14	Precipitation of the driest month	1.4	2.9
CEC	Cation exchange capacity	1.4	1.3
Bio8	Mean temperature of the wettest quarter	1.4	1.5
TEXCLS	Soil texture class	1.4	0.7
Thickness	Soil thickness	1.3	1.3
Bio17	Precipitation of the driest quarter	1.2	5.3
TP	Total phosphorus	0.8	0.8
TK	Total potassium	0.7	2.6
Bio2	Diurnal range mean	0.4	1.4
Bio9	Mean temperature of the driest quarter	0.3	0.5
BD	Bulk density	0.3	0.9
Bio16	Precipitation of the wettest quarter	0.1	0.6
Bio6	Minimum temperature of the coldest month	0.1	0.3
Bio11	Mean temperature of the coldest quarter	0	0
Bio12	Mean annual precipitation	0	0
Bio1	Mean annual temperature	0	0

Then, ENMtools was used to conduct correlation analysis of environmental variables, and R 4.3.2 (R Core Team, R Foundation for Statistical Computing, Vienna, Austria) was used to create a correlation heatmap (**Figure 2**). For the variables with a high correlation coefficient (|*r*| ≥ 0.8), only one representative variable with a great contribution was retained. High permutation importance indicates that the model is highly dependent on the variable (Phillips et al., 2006) For example, the permutation importance of Bio13 and Bio15 reached 8.1 and 10.4, respectively. Therefore, variables with both high correlation and high permutation importance were also retained. The source plants of FA prefer warm and humid environments, with weak cold tolerance. The preferred pH is slightly acidic to neutral, and the favorable soil type is loose and fertile sandy loam with good drainage (Jiangxi Provincial Market Supervision Administration, 2020). Accordingly, TEXCLS, pH, SLO, minimum temperature of the coldest month (Bio6), and precipitation of the warmest quarter (Bio18) could have prominent influence on the geographical distribution of FA.

Based on variable correlation, contribution to FA distribution, and plant growth habits, 16 environmental variables were selected for model construction. There were three topographic variables (ELE, SLO, ASP), eight bioclimatic variables (Bio5, Bio7, Bio4, Bio13, Bio15, Bio3, Bio6, Bio18), and five soil variables (CF, SOC, TN, TEXCLS, pH).

### 2.5 Construction of maximum entropy model

The geographical distribution data of FA and the data of selected environmental variables were imported into Maxent 3.4.4 (https://github.com/mrmaxent/Maxent) for modeling. The habitat suitability ranges from 0 to 1, where values closer to 1 indicate greater probability of species existence (Chen et al., 2024). The potential distribution areas of FA in Jiangxi were revealed using logistic equation. Based on the method of Moreno et al. (2011), 25% of the distributed data were randomly selected as the testing set and the remaining 75% as the training set. Response curves were derived to analyze the suitable ranges of environmental variables for FA, with the variable value as the horizontal coordinate and the distribution probability of FA as the vertical coordinate. Generally, the variable value corresponding to a distribution probability >0.5 is considered suitable for plant growth (Huan et al., 2023). Variable weights were determined by jackknife test (Li X. et al., 2023). Feature selection and parameter estimation were conducted using the bootstrap method (Lei and Chen, 2023), with 10 iterations. The maximum number of background points was 10,000 and the regulation multiple was 1, with other parameters set by default. The results of 10 runs were averaged and saved as an ASCII file (Soilhi et al., 2022).

### 2.6 Model accuracy evaluation

The accuracy of model predictions was assessed in terms of the area under the ROC curve (AUC), which is currently regarded as the optimal evaluation indicator. As the AUC value is not affected by diagnostic thresholds and not sensitive to species incidence, it can be used to compare the prediction accuracy of different models. An AUC value closer to 1 indicates better prediction performance of the model and greater influence of the environmental variables on the probability of crop suitability (Fielding and Bell, 1997).

## 3 Results

### 3.1 Environmental background of the study area

The environmental characteristics of FA sampling sites exhibit typical subtropical cultivation conditions, demonstrating that optimal growth requires: (i) sufficient hydrothermal conditions, (ii) well-drained terrain, and (iii) balanced soil fertility (**Table 2**).

**Table 2:** Basic information of climate, topography, and soil conditions across the sampling points of Fructus Aurantii in Jiangxi Province.

Variable	Mean	Minimum	Maximum	Skewness	Kurtosis	Most frequent value	Least frequent value
Bio1 (°C)	17.81	14.44	20.21	0.11	3.85		
Bio12 (mm)	1562.24	1434.00	1748.00	0.63	0.54		
ELE (m)	111.52	18.00	1163.00	5.74	42.77		
SLO (°)	1.06	0.02	6.52	2.45	6.64		
TEXCLS						Silt loam	Clay
SOC (g/kg)	4.93	3.01	7.13	0.46	0.33		
TN (g/kg)	0.58	0.49	0.74	0.75	0.1		
TP (g/kg)	27.69	20.00	39.00	0.63	0.21		
TK (g/kg)	14.22	10.02	18.68	0.01	0.77		

### 3.2 Prediction accuracy of the MaxEnt model

**Figure 3:**
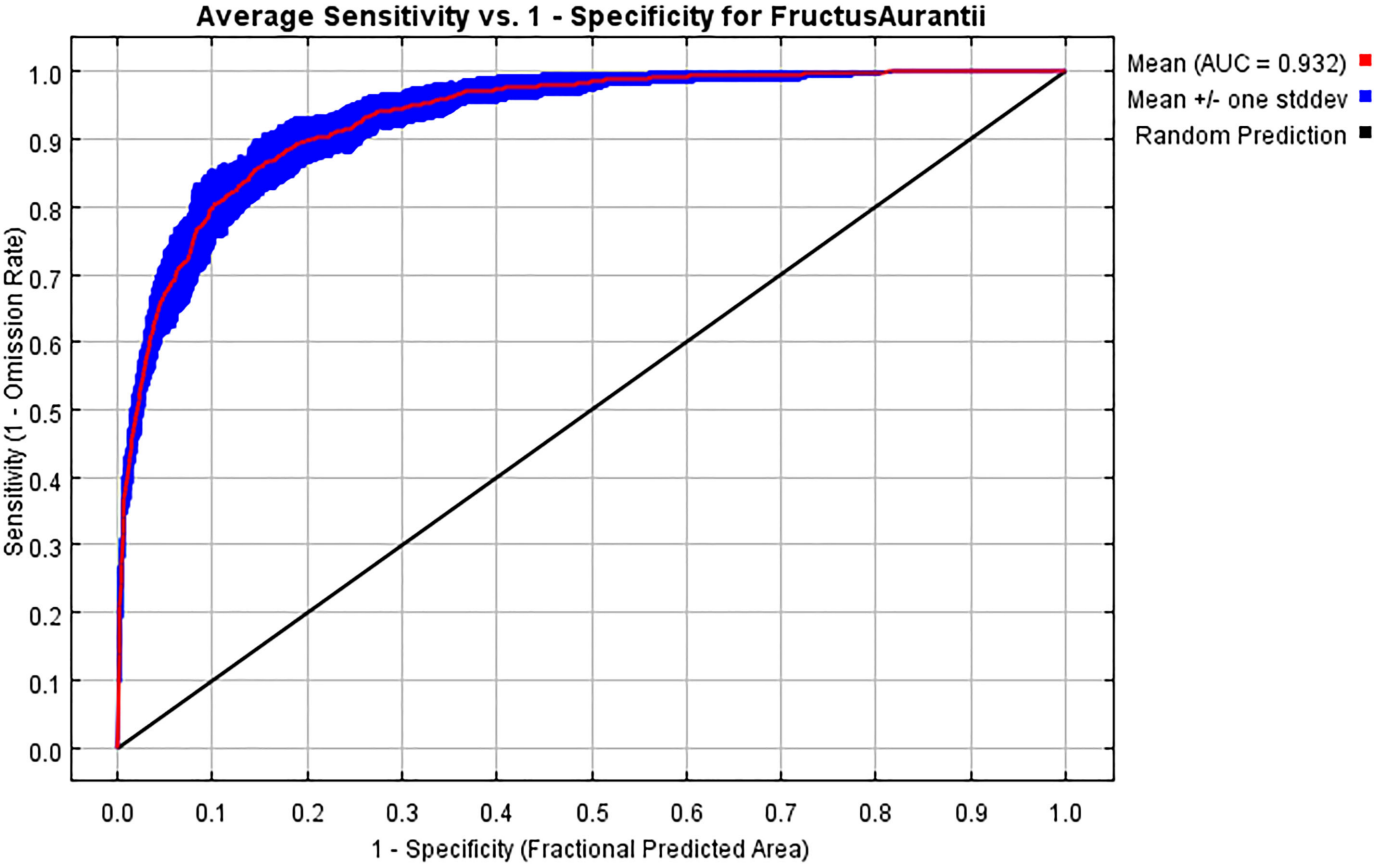
Prediction accuracy of the MaxEnt model for the distribution of Fructus Aurantii verified by the receiver operating characteristic curve.

The MaxEnt model demonstrated high predictive accuracy with an AUC of 0.932 (**Figure 3**), a kappa statistic of 0.82, and a test omission rate of only 7.83% under the minimum training presence threshold, confirming its reliability for predicting the distribution of FA.

### 3.3 Factors affecting the distribution of Fructus Aurantii and their suitable ranges

**Figure 4:**
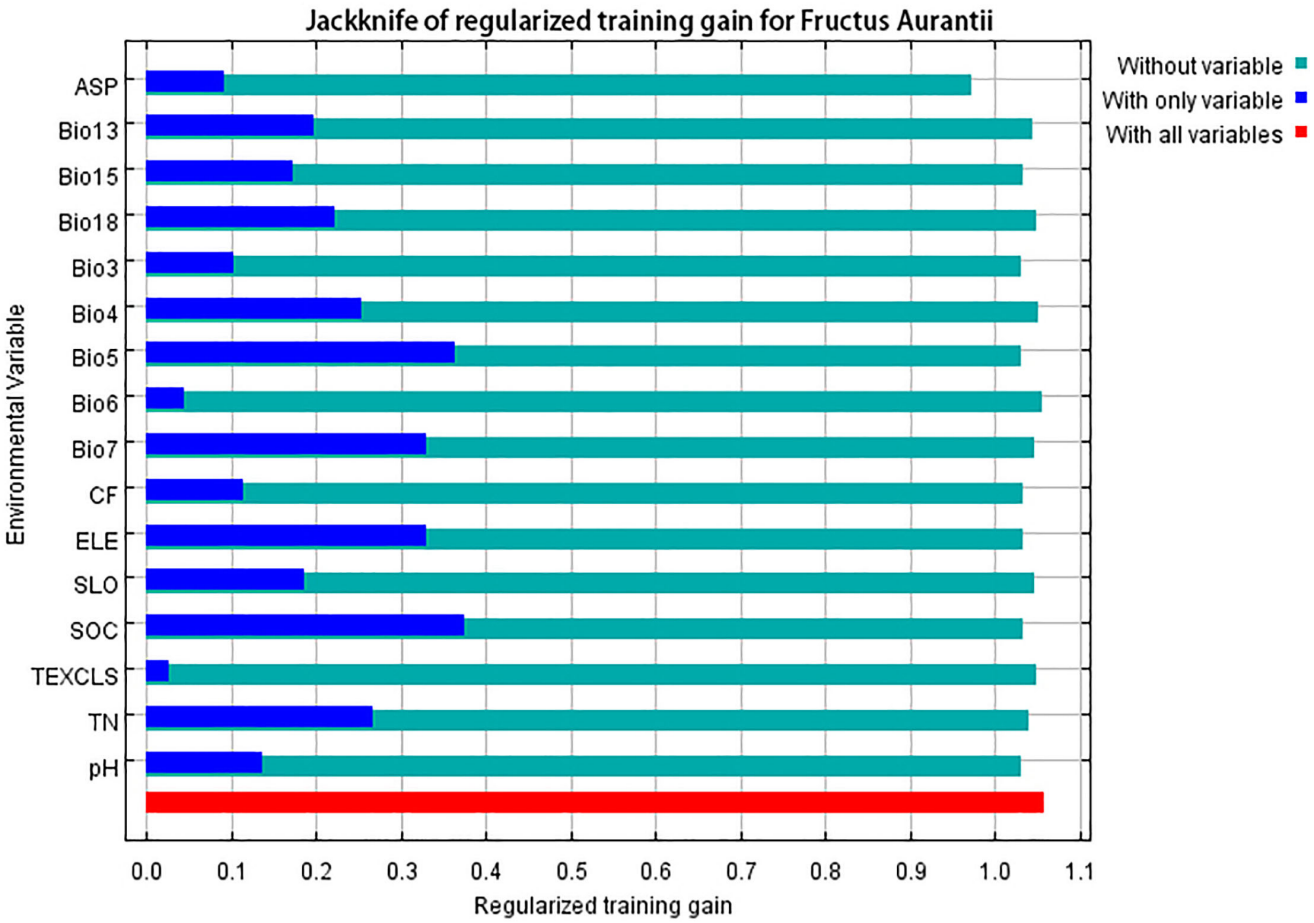
The results of Jackknife test for 16 environmental variables affecting the distribution of Fructus Aurantii.

**Figure 5:**
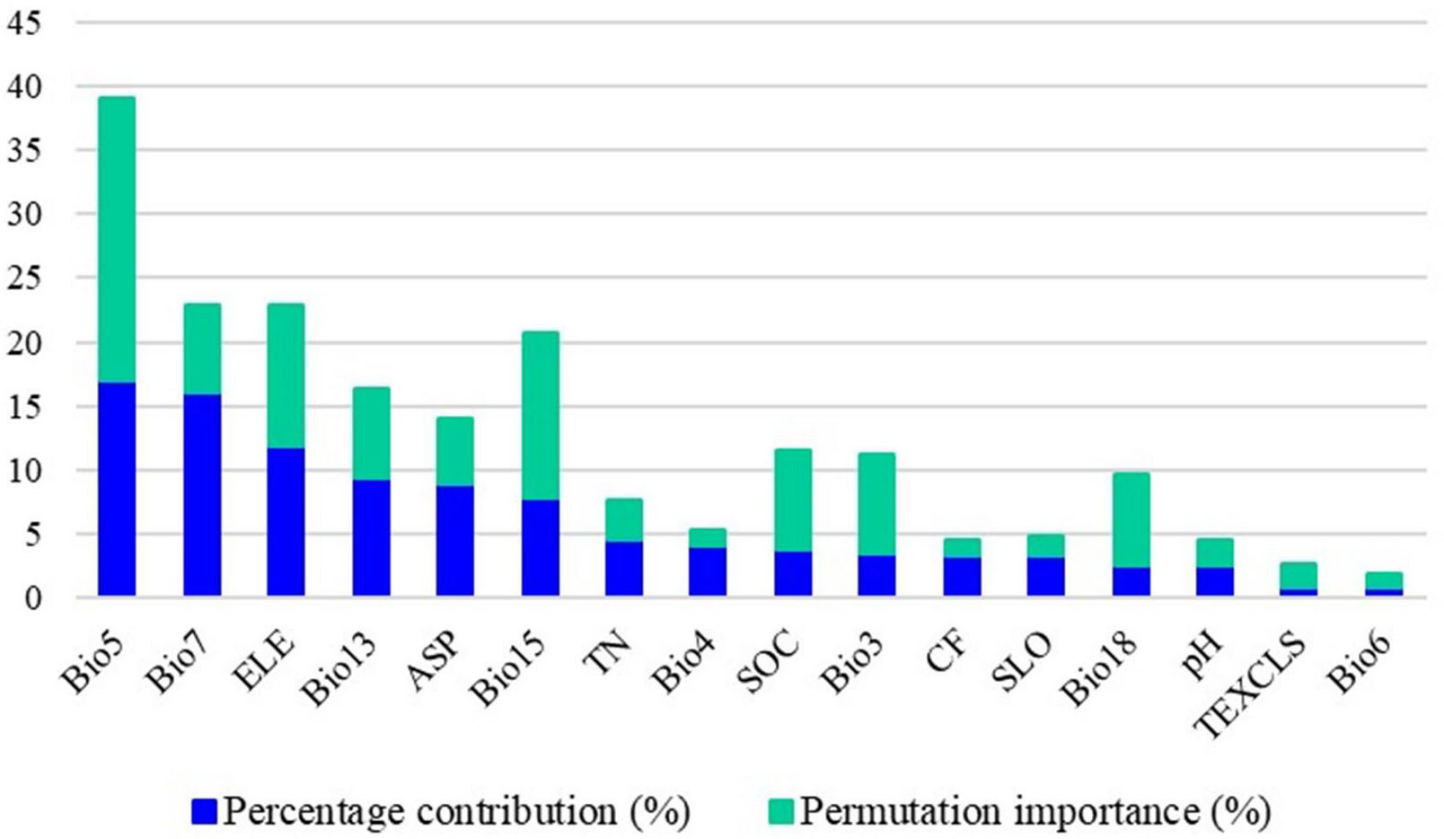
Percentage contribution and permutation importance of 16 selected environmental variables to the MaxEnt model for the distribution of Fructus Aurantii.

In the MaxEnt model, three methods or metrics—Jackknife test, percentage contribution, and permutation importance—are commonly used to evaluate the importance of environmental variables on species distribution. The Jackknife test of regularized training gain for FA revealed the importance of 16 environment variables on the predictive ability of the model (**Figure 4**). When used in isolation, the single variables with the highest gain were Bio5 and SOC, followed by Bio7 and ELE; the lowest gain was observed with TEXCLS. When removing a specific variable (e.g., ASP), a notable reduction in the gain indicated strong influence of this variable in the model. The major variables that contributed to model predictions in descending order were Bio5, Bio7, ELE, Bio13, ASP, and Bio15, with a cumulative contribution of 70.5% (**Figure 5**). The permutation importance corresponding to these major variables was also prominent, with a cumulative value of 65.2%. Other variables contributed less to the geographical distribution of FA. Among the environmental variables, bioclimate had the greatest influence on the geographical distribution of FA in Jiangxi (61% contribution to the model), followed by topography (24%) and soil (15%). Specifically, the principal bioclimatic variables were Bio5, Bio7, Bio13, and Bio15; the dominant topographic variables were ELE and ASP; the primary soil variable was SOC.

**Figure 6:**
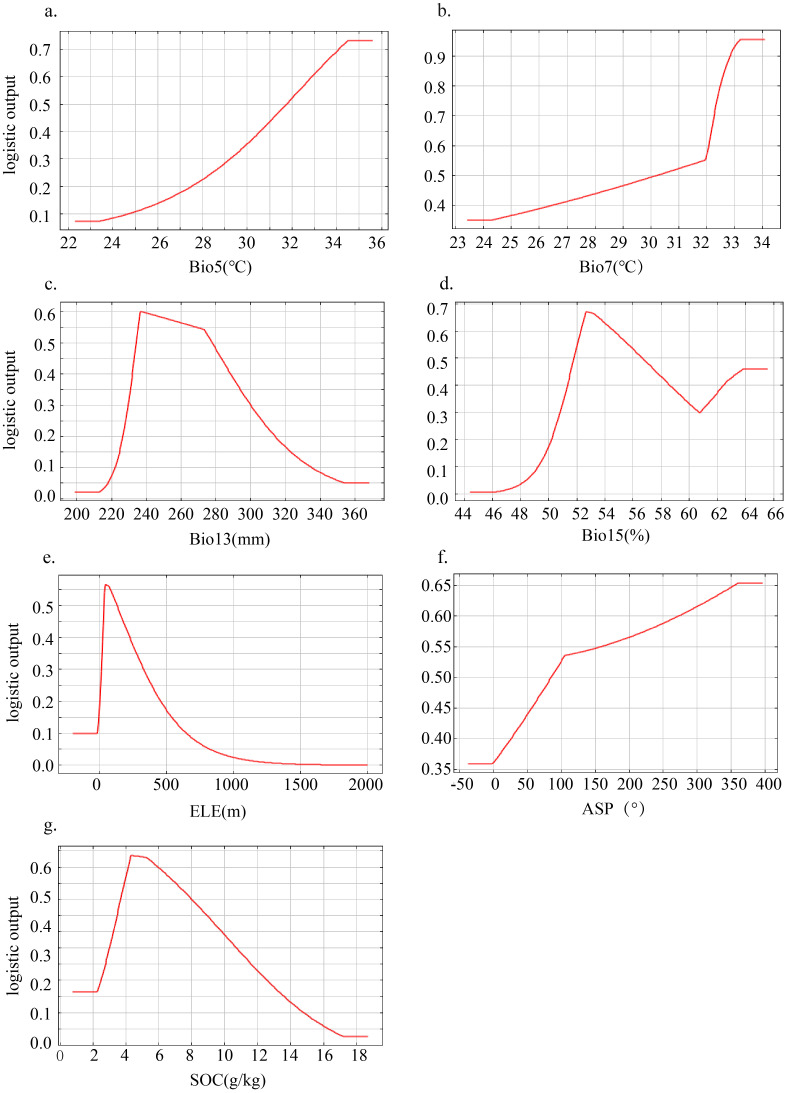
Response curves of the distribution probability of Fructus Aurantii to major environmental variables **(a–g)**.

The suitable ranges of major environmental variables affecting the geographical distribution of FA were determined based on the response curves. A peak value was observed for the distribution probability of FA when the environmental variables reached a certain level (**Figure 6**). For example, the distribution probability of FA trended upward rapidly with increasing ELE, Bio13, Bio15, and SOC in a narrow range, and then decreased after reaching a maximum (**Figures 6c–e, g**). Additionally, the distribution probability of FA increased slowly with elevating Bio5 and Bio7 until it reached a stable plateau (**Figures 6a, b**). FA showed certain adaptability to the seven major environmental variables, with the thresholds of 32–35°C for Bio5, 30–34°C for Bio7, 25–150 m for ELE, 235–258 mm for Bio13, (southeast, southwest, and northwest) for ASP, 52–56% for Bio15, and 4–8 g/kg for SOC.

### 3.4 Potential distribution of Fructus Aurantii under current climate conditions

**Figure 7:**
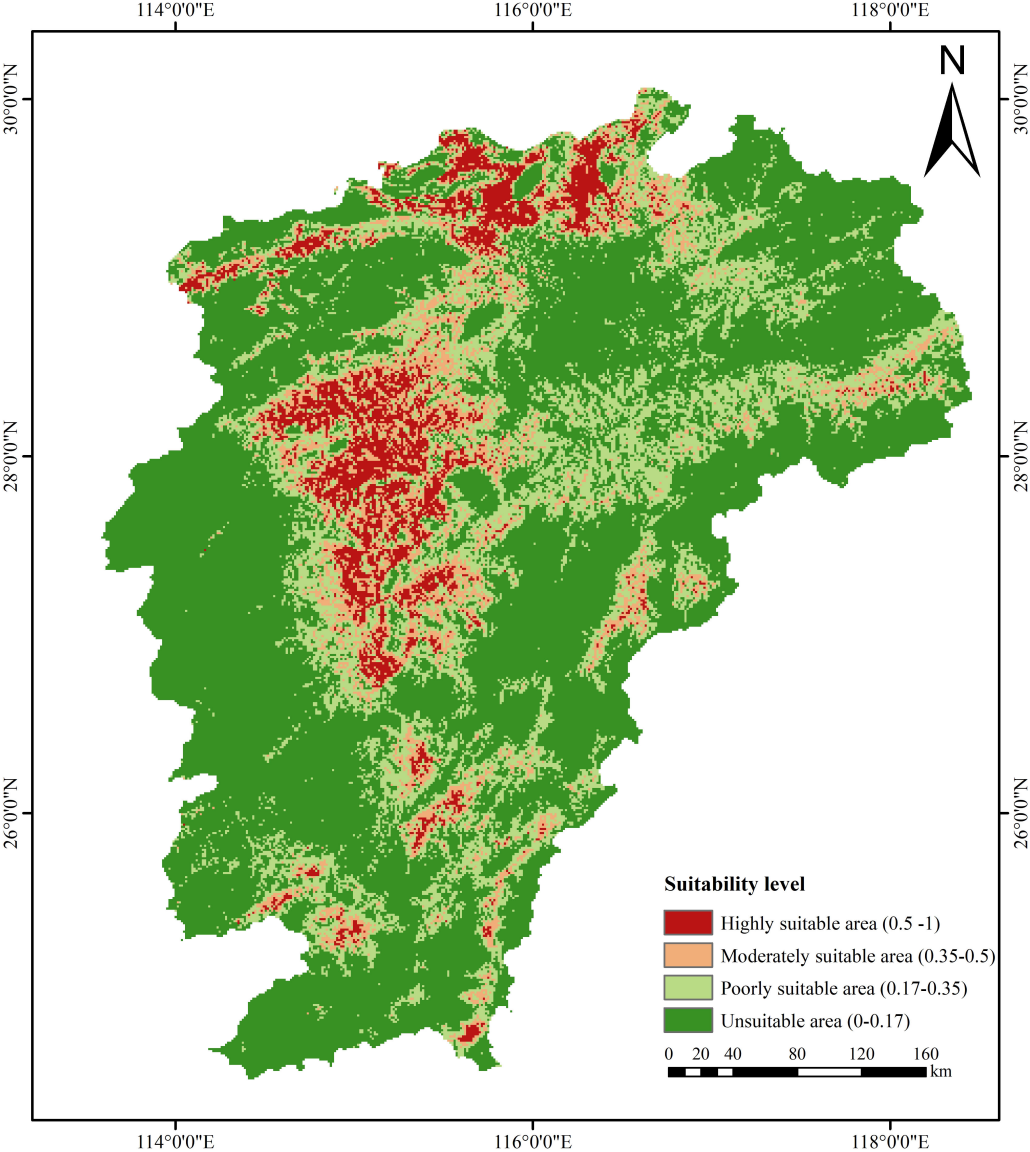
Distribution of habitat suitability for Fructus Aurantii in Jiangxi Province under the current climate scenario.

The predicted habitat suitability of FA in Jiangxi ranged from 0 to 0.91 under the current climate scenario, with the sampling points mainly distributed in highly suitable areas. This indicates that the MaxEnt-based prediction results of habitat suitability are basically consistent with the actual FA distribution. Based on the suitability values derived from actual FA distribution points and the predefined ecological factor thresholds, FA habitat suitability was classified into four suitability levels: unsuitable (0–0.17], poorly suitable (0.17–0.35], moderately suitable (0.35–0.5], and highly suitable (0.5–1]. Validation confirmed that most FA occurrences fall within suitable zones, and the resulting spatial distribution pattern aligns with the results obtained by Gao (2021). The total suitable area for FA was 6.30×10^4^ km^2^, accounting for 37.74% of the total area of Jiangxi (**Figure 7**; **Table 3**). Highly suitable areas (1.07×10^4^ km^2^) were primarily concentrated in eastern Jiujiang, southeastern Yichun, northeastern Ji’an, Xinyu, and Xingguo (a county in Ganzhou). Moderately suitable areas (1.44×10^4^ km^2^) were principally distributed in eastern Jiujiang, southeastern Yichun, northeastern Ji’an, northwestern Shangrao, southeastern Xinyu, and Xingguo. Poorly suitable areas (3.80×10^4^ km^2^) were less distributed in Pingxiang, with broader distributions in Jiujiang and Nanchang.

**Table 3:** Suitable area for Fructus Aurantii in Jiangxi under five different climate scenarios.

Climate scenario	Suitability level (10^4^ km^2^)				
	Unsuitable area	Poorly suitable area	Moderately suitable area	Highly suitable area	Total suitable area
Current	10.39	3.79	1.44	1.07	6.30
SSP245 2050s	3.06	5.72	5.45	2.46	13.63
SSP245 2070s	2.68	6.91	4.78	2.32	14.01
SSP585 2050s	1.96	7.58	5.38	1.77	14.73
SSP585 2070s	3.27	8.76	4.01	0.64	13.42

### 3.5 Potential distribution of Fructus Aurantii under future climate conditions

Climate-induced change of suitable distribution areas for genuine medicinal materials is likely to promote the transfer of genuine production areas or even the loss of genuineness. The existing genuine producing areas of FA in Jiangxi are particularly affected by climate change, as indicated by the results of the MaxEnt model that Bio5, Bio7, Bio13, and Bio15 had overarching effects on FA distribution. These bioclimatic variables directly relate to plant growth cycle and survival boundary, while indirectly affecting plant water acquisition and drought tolerance. In the wake of global warming, changes in these key bioclimatic variables may reshape the distribution of suitable planting areas for FA, altering habitat suitability in existing genuine producing areas.

**Figure 8:**
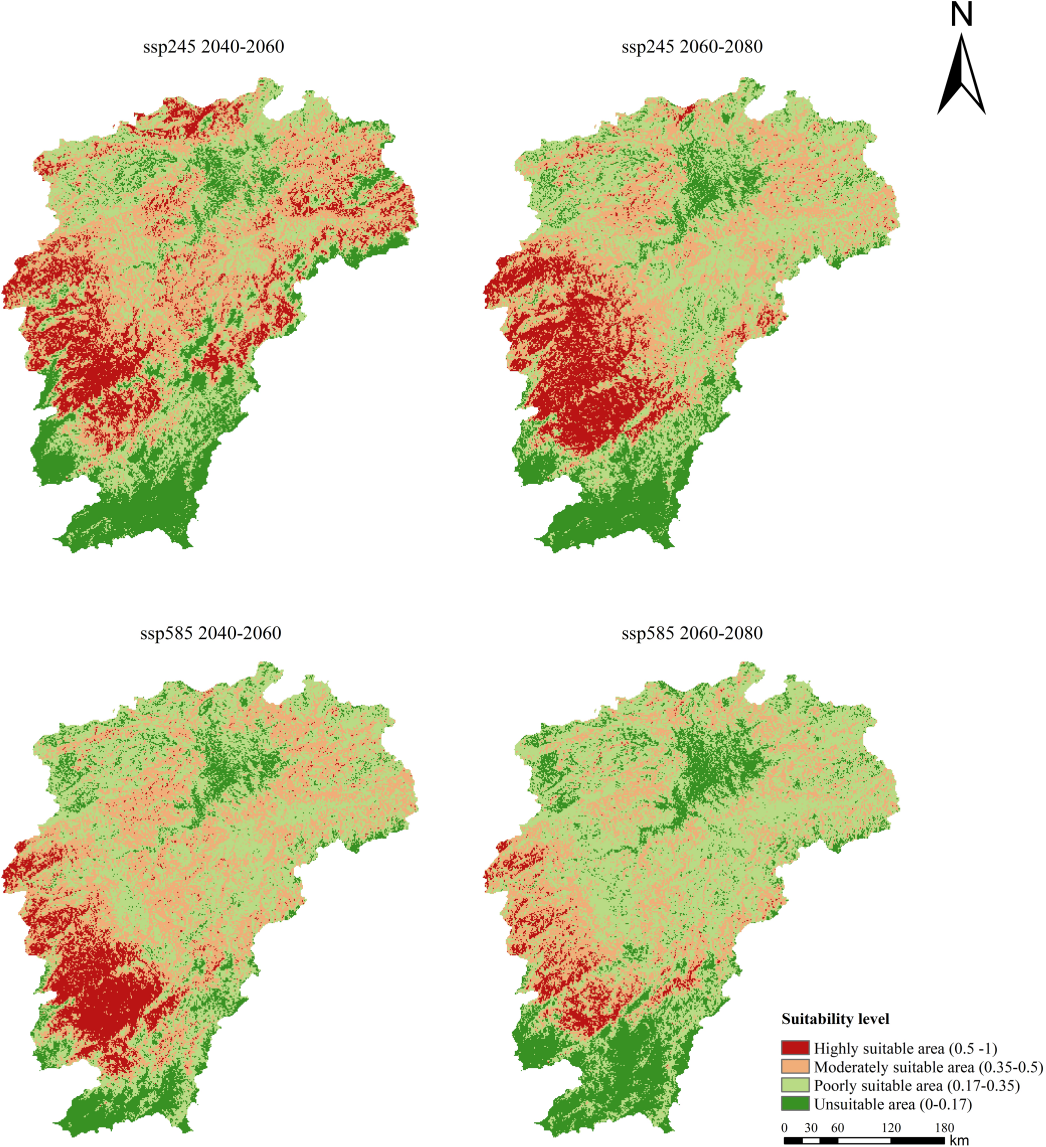
Distribution of habitat suitability for Fructus Aurantii in Jiangxi Province under different future climate scenarios.

Under the SSP245 scenario, the distribution range of FA was projected to shift southward from 2040 to 2080 (**Figure 8**). The total suitable area for FA would increase to 13.63×10^4^ km^2^ by 2060 and 14.01×10^4^ km^2^ by 2080 (**Table 3**). In terms of land quantity, the expansion of suitable area for FA is more evident over a longer time, despite at a lower growth rate. Under the SSP585 scenario, the distribution range of FA would also shift southward from 2040 to 2080. The total suitable area of FA would reach 14.73×10^4^ km^2^ by 2060 and 13.42×10^4^ km^2^ by 2080, indicating its considerable expansion followed by slight shrinkage. This expansion of suitable area for FA is primarily attributed to the increase in moderately and poorly suitable areas. Under both climate scenarios, the future suitable area for FA is more than double the current suitable area predicted by the MaxEnt model (**Figure 7**).

The results indicate that the suitable area for FA will expand in the future. Especially under the SSP245 scenario, the highly suitable area will prominently expand, almost double the current suitable area. With regard to the direction of spatial expansion, the suitable area will move southward mainly due to topographic regulation of local microclimate through the redistribution of water and heat. For example, the terrain of Ji’an is generally inclined from the south to the north, with relief rises in the south, low flat areas in the central part, and flat areas in the north. The highly suitable area in Ji’an will transfer from the northeastern part to the southwestern part in the future, corresponding to the topographic features. Shangrao has a terrain that is high in the southeast and low in the northwest, where the southeastern region will transform from moderately to highly suitable area in the future.

### 3.6 Planting regionalization for Fructus Aurantii considering future climate change

The regionalization of genuine medicinal materials is based on systematic investigation into the natural distribution patterns of genuine medicinal materials and the regional geographical features of their production areas. Effective regionalization of genuine medicinal materials can be achieved taking into account within-region similarity and between-region difference. Disentangling the relationship between the quality of genuine medicinal materials and the ecological environment of their production areas is essential for clarifying the specific ecological requirements of source plants and the mechanisms of environmental impacts. Such research also provides fundamental data for the regionalization and production layout of genuine medicinal materials. The core goal of regionalization is to reveal regional differentiation in the resources and production activities of genuine medicinal materials.

**Figure 9:**
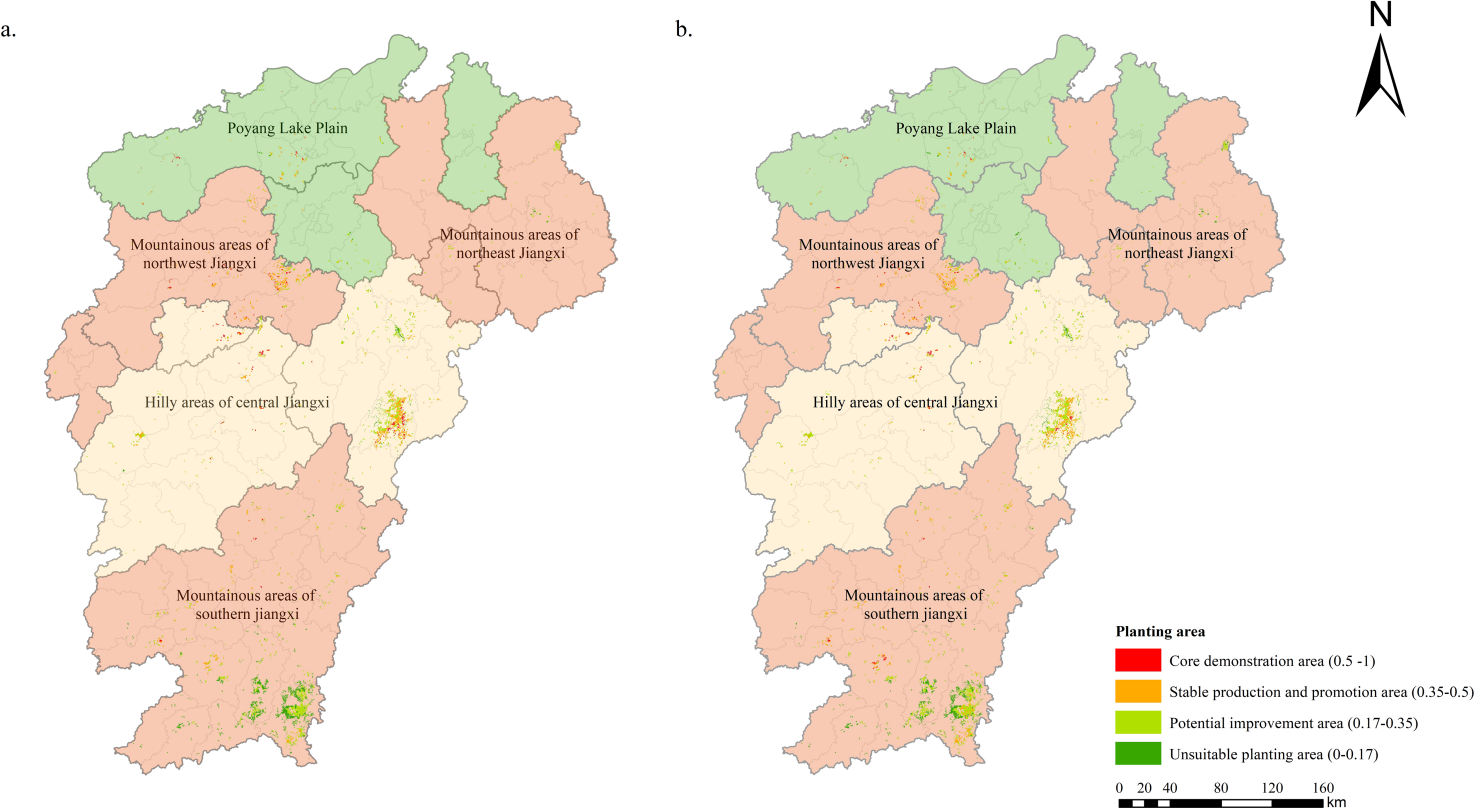
Planting regionalization of Fructus Aurantii in Jiangxi Province based on MaxEnt predictions of current and future distribution areas [**(a)** SSP245 scenario; **(b)** SSP585 scenario].

Given the limitation of land use, this study selected garden land polygons in Jiangxi as the basic evaluation unit to build a comprehensive regionalization system for FA that takes into account the influence of multiple environmental variables. A total of 996 qualified garden land polygons (1913.21 km^2^ in total) were selected after removing highly fragmented polygons (<4000 m^2^ each). Urban development boundaries, ecological protection red lines, and permanent prime farmland protection lines were also excluded. A weighted overlay analysis was conducted on the predicted current and future distribution areas for FA and the garden land polygons in Jiangxi. Based on the prediction results under current climate conditions and future climate change in the medium (2040–2060) and long (2060–2080) terms, different weights (0.5, 0.3, and 0.2, respectively) were assigned for comprehensive weighted assessment. Under the SSP245 scenario, the total suitable planting area for FA was estimated to be 1438.54 km^2^, accounting for 75.19% of the total garden area in Jiangxi. Under the SSP585 scenario, a slight increase was observed in the total suitable planting area for FA (1531.55 km^2^, 80.05%; **Figure 9**). The results indicate that the vast majority of garden land in Jiangxi can be actually planted for FA in the future.

Based on the habitat suitability of FA (from high to low level), the potential planting areas in Jiangxi can be divided into three parts: core demonstration area, stable production and promotion area, potential improvement area, and unsuitable planting areas (**Table 4**). Under the SSP245 scenario, the core demonstration area in Jiangxi was estimated to be 106.82 km^2^, mainly located in the central hilly area, northern plain area (Poyang Lake Plain), and southern mountainous area. The stable production and promotion area (518.59 km^2^) was chiefly found in the central hilly area and northern plain area, with the potential improvement area (813.13 km^2^) emerging in the northern plain area and southern mountainous area. Under the SSP585 scenario, the core demonstration area was estimated to be 82.19 km^2^, mostly distributed in the central hilly area. The stable production promotion area (539.31 km^2^) was mainly observed in the central hilly area and southern mountainous area, with the potential improvement area (910.05 km^2^) located in the central hilly area and southern mountainous area.

**Table 4:** Planting area of Fructus Aurantii in garden land of Jiangxi Province based on MaxEnt predictions under current climate conditions and two future scenarios for 2040–2080 (unit: km^2^).

Administrative region	Mountainous area of northeastern Jiangxi	Mountainous area of northwestern Jiangxi	Mountainous area of southern Jiangxi	Hilly area of central Jiangxi	Northern plain area of central Jiangxi (Poyang Lake Plain)	Total garden land area
SSP245						
Core demonstration area	4.18	5.85	20.84	38.21	37.74	106.82
Stable production and promotion area	27.59	16.94	80.26	174.18	219.62	518.59
Potential improvement area	42.68	106.50	258.06	151.84	254.05	813.13
Unsuitable planting area	18.40	143.16	225.94	35.52	51.66	474.67
Total suitable planting area	74.45	129.29	359.16	364.23	511.41	1438.54
SSP585						
Core demonstration area	0.00	18.36	18.65	34.48	10.71	82.19
Stable production and promotion area	4.30	114.89	160.64	225.60	33.88	539.31
Potential improvement area	45.39	67.96	402.98	354.87	38.85	910.05
Unsuitable planting area	17.28	5.58	298.87	43.73	16.20	381.66
Total suitable planting area	49.68	201.21	582.26	614.96	83.44	1531.55

## 4 Discussion

### 4.1 Major environmental factors driving the distribution of Fructus Aurantii

In China, the diverse and dynamic geo-ecological environment fosters the development of various ecotype patterns for genuine medicinal materials (Wu, 2004). Deciphering the interactions between genuine medicinal materials and their ecological environment is a popular topic in the field of traditional Chinese medicine. The quality of genuine medicinal materials is determined by multiple environmental factors that have complex coupling with each other. While the key environmental factors include sunshine, temperature, humidity, and soil properties, the major variables affecting the geographical distribution of genuine medicinal materials vary considerably across spatial scales. For example, water and heat conditions are regarded as the key factors dictating the distribution of genuine medicinal materials on a global scale, whereas local environmental variables (e.g., elevation, soil properties) emerge as the prominent drivers on a landscape or smaller spatial scale (Shang et al., 2005).

Our MaxEnt simulation results show that a set of bioclimatic variables (temperature: Bio5, Bio7; precipitation: Bio13, Bio15) play an overarching role in affecting the geographical distribution of FA in Jiangxi. The territory of Jiangxi spans multiple degrees of latitude, resulting in a large temperature gradient. As the source plants of FA are sensitive to cold damage, temperature emerges as the leading driver of FA distribution (Zabihi et al., 2016). The suitable range of Bio5 (maximum temperature of the warmest month) for FA is 32–35°C. Beyond this temperature range, especially high temperature before fruit harvest may lead to rapid pulp enlargement, peel thinning, and even premature ripening as well as peel shrinkage. Heat stress could also interfere with plant secondary metabolism, affecting the accumulation of effective components and resulting in declined quality of FA (Yang et al., 2008). Bio7 (annual temperature range) in the range of 30–34°C is suitable for FA. Generally, plant growth cycle is disrupted upon intensified temperature variations. FA is derived from perennial shrubs, whose shoots can be damaged by low temperature (Tu, 2018), increasing the risk of pest invasion and impeding root water uptake.

With regard to precipitation, Bio13 (precipitation of the wettest month) in the range of 235–258 mm is suitable for FA. Excessive precipitation is likely to cause root rot, as well as flower and fruit drop (Wei, 2009). From the perspective of topography, FA is suitable to be distributed in the ELE range of 25–150 m asl, which facilitates drainage. The suitable ASP conditions include the southeast, southwest, and northwest, providing sufficient lighting. Despite relatively low importance of soil variables to the distribution of FA, attention should be paid to plant preference for slightly acidic to neutral loam or sandy loam.

### 4.2 Future distribution pattern of suitable planting areas and regionalized strategies

Based on the MaxEnt prediction results, the suitable area for FA covers 6.30 × 10^4^ km^2^ of garden land under the current climate conditions, mainly in the northern part of central Jiangxi. Numerous studies have underscored the role of temperature as a key environmental factor limiting species distribution on the latitude gradient (Qian and Ricklefs, 2011). Global warming exhibits a positive effect on the distribution and expansion of thermophilous plants (Zheng and Cao, 2020). Temperature rise can increase the accumulated temperature during plant growth period, consequently promoting plant growth and increasing plant biomass (Baldwin et al., 2014). Under different future scenarios of global warming, there are notable changes in the major environmental variables affecting the geographic distribution of FA. Bio5 is expected to increase by ~5.5°C and 6.4°C under SSP245 and SSP585 scenarios, respectively, corresponding to ~2.9°C and 2.6°C increase in Bio7. As such, the future distribution range of FA is predicted to expand twice its current size.

Among the topographic factors, ELE has a profound impact on the distribution of FA in the study area. Topography also contributes greatly to spatial heterogeneity in regional climate change, thus affecting the response of vegetation to climate (Chang et al., 2015; Xiong et al., 2023). The terrain of Jiangxi mainly encompasses mountains in the east, west, and south; there are hills alternating with river valley plains in the central part, whereas the Poyang Lake Plain lies in the north. Due to this high south and low north pattern, FA populations are likely to migrate southward to cooler areas at higher elevations, as driven by future extreme heat. However, future temperatures will increase substantially, leading to more frequent climate extremes (e.g., droughts, heat waves, rainstorms) and impairing the soil carbon sink function (Melillo et al., 2017). Under these adverse environmental changes, the potential suitable area for FA may diminish under the SSP585 climate scenario of extreme warming in 2080. Complex plant physiological and ecological responses to climate change affect the potential distribution pattern of FA in the future (Li et al., 2009). This study uncovers that future climate warming will drive the expansion of potential suitable areas for FA in Jiangxi; however, excessive warming may lead to the reduction of highly suitable areas in the future, affecting the medicinal properties and germplasm of FA. Therefore, more effort should be dedicated to the preservation of seed sources and breeding for improved plant varieties to cope with the risk of future extreme heat.

Taking into account the potential dynamic change of future climate, we prospectively regionalize the suitable planting areas for FA into three parts and propose tailored development strategies. i) In the core demonstration area, the conventional planting industry of FA should be preserved and optimized. On this basis, it is suggested to continuously introduce advanced cultivation techniques and integrated pest control strategies, as well as formulate strict quality control standards and operating specifications. Moreover, it is crucial to actively breed for heat-resistant plant varieties and establish an excellent germplasm resource bank, which can ensure and improve the medicinal quality of FA. ii) In the stable production and promotion area, the existing management practices and the technical levels of farmers should be improved to maintain the stable and high yield of FA. iii) In the potential improvement area, it is recommended to appropriately expand the planting areas for FA based on market demand, and select the plant varieties with strong adaptability taking into account regional microclimate characteristics. Furthermore, the investment in infrastructure construction needs to be increased and the irrigation, drainage, and transportation conditions should be improved in order to reduce the harm of natural hazards and ensure the quality of FA.

### 4.3 Applicability and limitations of maximum entropy model

As Chinese medicinal materials are characterized by unique geographical distribution patterns, it is unrealistic to analyze their ecological space requirements by physiological measurements. The MaxEnt model uses multiple environmental variables of known species distribution points to simulate species distribution and then project it into another geographical space. Zeng et al. (2021) identified the key environmental variables affecting the cultivation of *Pogostemon cablin* Benth and predicted its future potential planting areas using the MaxEnt model combined with ArcGIS 10.2. Yan et al. (2024) conducted meta-analysis and MaxEnt modeling to uncover the impacts of global climate change on the quality and distribution of *Panax*. Li et al. (2024) predicted the spatial distribution of three *Ephedra* species under climate change based on the MaxEnt model. Given its high accuracy and practical application performance, MaxEnt is advantageous over other ecological niche models for predicting the potential distribution of Chinese medicinal materials (Zhu et al., 2013).

In this study, mean annual data were used to evaluate the impacts of climate on FA distribution, making it difficult to accurately reflect the similarity in variable response curves during the plant growth cycle of genuine medicinal materials. There were also limitations in describing the similarity among samples, as the data were unable to fully capture complex ecological characteristics. More advanced measurement methods should be explored to enable accurate assessment of ecological similarity across regions. Additionally, the BCC-CSM2-MR model was used in MaxEnt-based prediction under future climate scenarios. The ability of this climate model to simulate land surface variables needs to be improved. Furthermore, the potential distribution areas usually represent environmental settings similar to the actual distribution areas, rather than the real distribution boundaries. MaxEnt modeling establishes a statistical relationship between habitat distribution and environmental variables. It assumes that species distribution relates to environmental variables only, without considering the phenotypic plasticity and diffusion dynamics of species. Therefore, it is necessary to assess potential species distribution and extinction risk by integrating the life history and distribution history of species, as well as the scenarios of future climate and land use change.

For the planting regionalization of FA, this study did not take into account environmental pollution (e.g., man-made pollution sources) or socio-economic factors (e.g., urban expansion), which could have potential impacts on genuine medicinal materials. Additionally, attention should be paid to economic and technical factors in actual cultivation. On the basis of field investigation, small-scale planting trials can be carried out first in accordance with local economic levels, cultivation techniques, and seed and seedling quality. Agricultural facilities (e.g., greenhouses, sheds) and practices (irrigation, soil amelioration, and plastic mulching) are useful to reasonably regulate local eco-environmental conditions before large-scale introduction and planting of FA.

## 5 Conclusion

This study predicted the potential suitable areas for Fructus Aurantii (FA) in Jiangxi Province under future climate change based on the maximum entropy model with high accuracy. The potential suitable areas for FA were mainly distributed in the central hilly area, northern plain area, and southern mountainous area of Jiangxi. Climate played an overarching role in shaping the geographical distribution of FA, followed by topography and soil factors. Model prediction indicated that the total suitable area would expand twice its current size and shift southward in 2040–2080. The medium greenhouse gas emission scenario (SSP245) was more favorable for FA than the high greenhouse gas emission scenario (SSP585). The protection of FA is a long-term process, where planting regionalization should take into account the impact of future climate change. This study presents empirical data for rational selection of planting sites for FA and provides a modeling tool for accurate prediction of suitable distribution areas for other Chinese medicinal materials.

## Data Availability

The datasets presented in this study can be found in online repositories. The names of the repository/repositories and accession number(s) can be found in the article/supplementary material.
